# Body Cognition and Self-Domestication in Human Evolution

**DOI:** 10.3389/fpsyg.2019.01111

**Published:** 2019-05-21

**Authors:** Emiliano Bruner, Ben T. Gleeson

**Affiliations:** ^1^Centro Nacional de Investigación sobre la Evolución Humana, Burgos, Spain; ^2^Fenner School of Environment and Society, Australian National University, Canberra, ACT, Australia

**Keywords:** brain evolution, parietal lobes, spatial cognition, association cortex, life-history, social evolution, extended cognition

## Domestication and Human Self-Domestication

The term “domestication syndrome” describes a range of correlated trait changes seen in domesticated populations when compared to their wild relatives or ancestors (Jensen, 2006; Wilkins et al., 2014; Zeder, 2015). Controlled experimental breeding has demonstrated rapid emergence of this syndrome in several mammal populations selected for dampened reactive aggression and stress response (Trut, 1999; Jensen, 2006; Kulikov et al., 2016). These results confirm findings of correlated change from longstanding observational research in domesticated lineages (Hemmer, 1990). Known traits include: docile behavior; reduced sexual dimorphism; reduced prognathism; smaller teeth; skeletal gracility; reduced brain sizes; altered oestrus cycles and fertility; floppy ears; elevated vocal communication; and altered pigmentation (Hemmer, 1990; Wilkins et al., 2014; Sánchez-Villagra et al., 2016; Okanoya, 2017). Many of these features are known to appear rapidly, as heterochronic shifts in ontogeny (i.e., paedomorphism or neoteny), rather than as isolated and adaptive mutations (Belyaev, 1979; Trut, 1999; Jensen, 2006; Zeder, 2012, 2015). Heritable hypoplasia of neural crest cell-derived tissues provides the most widely supported proximate explanation for these observed trait correlations (Wilkins et al., 2014).

Interestingly, several traits seen in bonobos (Hare et al., 2012) and in humans (Groves, 1999; Leach, 2003; Cieri et al., 2014; Thomas and Kirby, 2018) suggest intraspecific interactions can drive a process of “self-domestication” via socio-sexual selection for higher social tolerance and less reactive aggression (Cieri et al., 2014; Hare, 2017; Wrangham, 2018). In *Homo sapiens*, this process is thought to have enabled an expanded cooperative ability, leading to improved language and knowledge-sharing, thereby promoting social complexity and technological advancement (Hare, 2017; Thomas and Kirby, 2018). Humans are also characterized by an outstanding capacity for integration between brain, body and tools, and the evolution of this ability is associated with neuroanatomical changes of the visuospatial association cortex (Bruner, 2018). Whilst current scholarship is yet to address the potential for interaction between self-domestication and body cognition, we hypothesize that there may be value in an examination of any overlap. As such, here, we consider whether and to what extent these phenomena shared common evolutionary factors or reciprocal influences.

## The Evolution of the Parietal Cortex in Humans

One of the main goals in evolutionary neurobiology is to identify features and aspects of the human brain that differ from other living and extinct primates (Preuss, 2017). When compared with extant taxa, *Homo sapiens* is characterized by cerebral features specific to our species, even if, for many of them, it is not clear whether they reflect simple differences in size (due to our peculiarly larger brain) or are entirely novel cerebral traits. Comparison with fossil hominids reveals differences in brain size, but a shared sulcal pattern and overall morphological organization (Bruner, 2017). Notably, there are differences in the cortical proportions of the parietal lobe, which shows dorsal regions that are wider in Neanderthals and generally much larger in modern humans (see Bruner, 2018 for a review). These regions spatially correspond to the precuneus and to the intraparietal sulcus, which have a larger and more complex cortical surface in humans when compared with other primates, including apes.

The parietal cortex is involved in multiple association tasks, but is particularly crucial for visuospatial integration—bridging body and vision, and coordinating eye and hand—and is central to functions like visual imaging, body-centered space and time simulation, and self-awareness (Fletcher et al., 1995; Cavanna and Trimble, 2006; Margulies et al., 2009; Freton et al., 2014; Land, 2014). These functions are also involved in relationships between brain and body and between body and environment; key factors that allow offloading and exporting of cognitive functions to external components (especially technology), thereby integrating tools into cognitive schemes of the body (Byrge et al., 2014; Bruner and Iriki, 2016).

**Figure 1:**
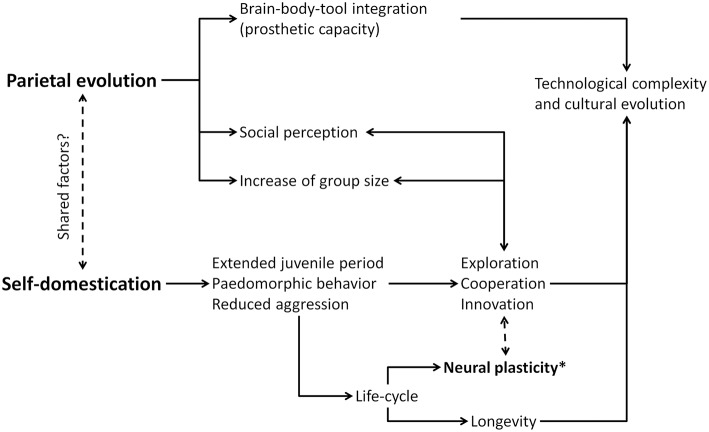
Diagram of possible relationships between parietal evolution and self-domestication. Neural plasticity (*) can be a key factor, being targeted by selective processes as to enhance cognitive, cultural, and social capacities.

Morphological changes in the modern human parietal cortex are not described among early *Homo sapiens* populations (say 100–300 thousand years ago), but are detected in later specimens, roughly at the time the archaeological record begins to show complex tools, projectile technology, and complex graphic culture (Bruner and Pearson, 2013; Neubauer et al., 2018). These developments, and this timeframe, have also been associated with reductions in masculine craniofacial morphology thought to indicate a process of human self-domestication (Cieri et al., 2014). If self-domestication was a crucial process in modern human evolution, and if body-tool extension and visual imaging have been key factors in modern human parietal cortex development, it makes sense to expect some interaction between their relative causes, effects, and functional mechanisms. As such, it appears worthwhile to consider whether these two features (self-domestication and visuospatial cognition) exert reciprocal influences and, further, whether these complex processes may share contributing factors in common (Figure 1).

## The Domesticated Brain

In general, brain size is substantially reduced in domesticated lineages when compared to non-domesticated forms (Kruska, 1988, 2005), and such reduction is more prominently expressed in more encephalized taxa (Kruska, 1988). Brain size reduction should, therefore, be particularly apparent in modern humans under the effects of domestication. However, although late modern humans display smaller cranial capacity when compared with earlier populations (Henneberg, 1988; McHenry, 1994; Ruff et al., 1997), relative brain size appears to have increased, because of a simultaneous reduction of body size (McHenry, 1994). We can wonder whether the novel expansion of derived areas (like the parietal ones) could have partially contrasted and masked a generalized reduction of brain volume in our species.

During domestication, limbic structures are particularly reduced (Kruska, 1988). This is probably crucial to achieve a lower aggressive reactivity and, accordingly, to promote and extend social bonds. However, presently available evidence suggests that humans have relatively larger—instead of smaller—limbic components (hippocampus, amygdala and orbito-frontal cortex), at least when compared with living apes (Barger et al., 2014). Such structures are, unfortunately, not directly detectable in fossil species, or in early modern humans.

## Juvenile Brains, Bodies, and Tools

Many non-human species utilize “objects” to some extent, but only humans use “tools,” as defined in a strict sense. Here, we suggest that to be “a tool,” an object must fulfill at least three crucial conditions. First, it must be integrated within the body schemes of the brain, as a real extension of its space and functions (Maravita and Iriki, 2004; Tunik et al., 2007; Heed et al., 2015). Second, it must be part of a productive chain, in which a propaedeutic sequence of tools is necessary to achieve a final target (Muller et al., 2017). Third, it must not simply *assist* the ecological and economical behavior of a species, but must be *integrated-with*, and *necessary-to*, a cultural niche (Plummer, 2004). Humans achieve these three conditions by integrating technology into cognitive processes, literally as a spider does with its silk web (Kaplan, 2012; Japyassú and Laland, 2017). According to theories in extended cognition, tools are proper functional elements of our cognitive system (Malafouris, 2010, 2013). That is, our cognitive process does not rely only on the neural system, but also on extra-neural components (technology) to which we delegate specific cognitive functions (Overmann, 2015). Such *prosthetic capacity* can be defined as the capacity to delegate cognitive functions to external elements, offloading and outsourcing information processing to peripheral (out-of-the-body) components. The parietal cortex in humans is involved in tool use and tool making (Grefkes and Fink, 2005; Bzdok et al., 2015; Goldring and Krubitzer, 2017; Kastner et al., 2017), and hence particularly involved in body-tool extension and integration (Bruner and Iriki, 2016). Human prosthetic capacity is largely enhanced by the remarkable plasticity of our cortical system (Sherwood and Gómez-Robles, 2017), and by the high level of creativity and explorative innovation of our species (Kyriacou and Bruner, 2011). Both features (neural plasticity and explorative behavior) are primarily associated with juvenile life stages and have been enhanced by extension of the juvenile period in humans (Bogin, 1990; Pellegrini et al., 2007). Given that animal domestication is broadly associated with a trend toward relative juvenilization (Harvey and Clutton-Brock, 1985; Smith, 1992; Joffe, 1997), aspects of human self-domestication may also contribute to our enhanced technological capacity. In fact, altered timing and stretching of the life-history is implicated in the extension of those ontogenetic stages more sensitive to novelty, the extension of the post-reproductive period, and the extension of life in general (longevity). All of these aspects of human life-history are strictly necessary to generate intergenerational transfer and cultural evolution (Kaplan and Robson, 2002; Lee, 2003), providing a further link between self-domestication and technological extension.

Interestingly, interpreting parietal expansion as an evolutionary novelty may complicate one diagnostic feature of the supposed juvenilization process in humans: that is, the roundedness of our head, which is often explained as a pedomorphic feature, but which could actually represent an apomorphic cortical character, mimicking a juvenile appearance. Apart from parietal bulging, vault globularity in our species is also due to the curvature of the frontal squama, likely to be a secondary structural consequence of having a reduced facial block positioned under the frontal lobes (Pereira-Pedro et al., 2017). This latter feature can indeed be associated with a pedomorphic process, at least if we consider the reduction of the splanchnocranium as a juvenile heterochronic retention.

## Association Cortex, Body Perception, and Social Evolution

A further potential locus of association between parietal expansion and self-domestication is increased sociability. The experimentally demonstrated proximate cause of domestication syndrome is selection against reactive, or autonomic, aggressive response (Trut, 1999; Jensen, 2006; Wilkins et al., 2014). This selective mechanism is thought to have facilitated the emergence of language, increased group sizes, and elevated cooperation in humans (Cieri et al., 2014; Hare, 2017; Thomas and Kirby, 2018). In primate species, group size is proportional to brain size and, for humans, it approaches 150 units (i.e., “Dunbar's number”—Dunbar, 2012, 2018). Interestingly, this correlation particularly concerns the association cortex, probably because of a direct relationship with behavioral complexity (Dunbar and Shultz, 2007; Pearce et al., 2013). The parietal cortex is one of the main association regions (Krienen and Buckner, 2017; Mars et al., 2017) and, in this case, its expansion is likely to have a direct effect of social group size. Moreover, the parietal cortex and visuospatial integration are involved in self-recognition, self-other perception, body-centered simulation, and in the management of a “social space” which uses the body as a functional and metric unit (Hills et al., 2015; Maister et al., 2015; Peer et al., 2015). Actually, the precuneus has been hypothesized to be a crucial element of the network involved in mind reading (Heyes and Frith, 2014). These features (increased social group size through increase of association functions, and the capacity to handle a social space based around one's own body) are strictly intermingled with social effects expected from self-domestication and associated juvenilization, namely an increase in the size and complexity of the social network.

## Conclusions

We hypothesize that, in humans, changes associated with self-domestication might have influenced, or been influenced by, body cognition, visuospatial integration, technological extension, and the evolution of the parietal cortex. Alternatively, these features may be independent, and might have evolved independently along the human lineage. These two hypotheses should be discussed and evaluated according to a comparative and functional perspective by investigating this possible association in other primates and considering the corresponding relationships between anatomy, development and cognition. Some aspects of these evolutionary features are likely to have interacted, generating reciprocal enhancement. Others may hide common mechanisms, possibly due to ontogenetic communalities and shared developmental components. In this regard, one candidate may be neural plasticity, which is both a crucial consequence of paedomorphic conditions and a feature particularly influencing the development of the parietal cortex because of its sensitivity to sensorial (somatic and visual) inputs. We can wonder whether sociability associated with self-domestication, an extended juvenile period, and increased neural plasticity, could have prompted the expansion of the parietal cortical surface, subsequently triggering retroactive feedback to enhance its functional consequences. Association cortices may be the result of multiple crossing gradients between sensorimotor regions, generating a patchwork of neural combinations in terms of functional properties (Huntenburg et al., 2017). In this case, prolonged or increased plasticity of the body-vision system may be the essential prerequisite for developing a more anatomically and functionally complex prosthetic capacity, as the ability to incorporate tools into body schemes, offloading cognitive processes to external elements. Importantly, it remains to be evaluated whether this process is strictly associated with the evolution of modern humans (*Homo sapiens*), or can be traced back to the origin of our genus. In any case, it seems important to consider these processes and functions together when attempting to determine a comprehensive evolutionary narrative for our species.

## Author Contributions

Both authors have made a substantial, direct and intellectual contribution to the work, and approved it for publication.

### Conflict of Interest Statement

The authors declare that the research was conducted in the absence of any commercial or financial relationships that could be construed as a potential conflict of interest.
